# Combining Blue Light and Yellow Curcumin to Obtain a “Green” Tool for Berry Preservation against Bacterial Contamination: A Preliminary Investigation

**DOI:** 10.3390/foods12102038

**Published:** 2023-05-18

**Authors:** Ilaria Stura, Zunaira Munir, Lorenza Cavallo, Luisa Torri, Narcisa Mandras, Giuliana Banche, Rita Spagnolo, Raffaele Pertusio, Roberta Cavalli, Caterina Guiot

**Affiliations:** 1 Department of Neurosciences, University of Turin, 10125 Torino, Italy; 2 Department of Public Health and Pediatric Sciences, University of Turin, 10126 Torino, Italy; 3 University of Gastronomic Sciences, 12042 Pollenzo, Italy; 4 Department of Drug Sciences and Technologies, University of Turin, 10125 Torino, Italy

**Keywords:** berries, food preservation, curcumin, photoactivation, β-cyclodextrin, antibacterial efficacy

## Abstract

*Background*: According to recent studies, tens of millions of tons of fruit are wasted each year in Europe in primary production and home/service consumption. Among fruits, berries are most critical because they have a shorter shelf life and a softer, more delicate, and often edible skin. Curcumin is a natural polyphenolic compound extracted from the spice turmeric (*Curcuma longa* L.) which exhibits antioxidant, photophysical, and antimicrobial properties that can be further enhanced by photodynamic inactivation of pathogens when irradiated with blue or ultraviolet light. *Materials and methods*: Multiple experiments were performed in which berry samples were sprayed with a complex of β-cyclodextrin containing 0.5 or 1 mg/mL of curcumin. Photodynamic inactivation was induced by irradiation with blue LED light. Antimicrobial effectiveness was assessed with microbiological assays. The expected effects of oxidation, curcumin solution deterioration, and alteration of the volatile compounds were investigated as well. *Results*: The treatment with photoactivated curcumin solutions reduced the bacterial load (3.1 vs. 2.5 colony forming units/mL (UFC/ml) in the control and treated groups; *p*-value = 0.01), without altering the fruit organoleptic and antioxidant properties. *Conclusions*: The explored method is a promising approach to extend berries’ shelf life in an easy and green way. However, further investigations of the preservation and general properties of treated berries are still needed.

## 1. Introduction

Around 28.1 million tons (Mt) of fruit are currently wasted each year in the EU [1,2], considering all the stages in the food supply chain (11.1 in primary production, 6.1 in processing, 0.8 in distribution, and 10.1 in consumption) [2]. The two more problematic steps are primary production and home (8.6 Mt) and service (1.5 Mt) consumption. Among fruits, berries are the most delicate, have a short shelf life, and easily rot before being consumed, undergoing water loss and mechanical damages along the production/marketing chain. Moreover, the presence of microorganisms can alter the aspect and taste of berries.

Many techniques are used to preserve fruits from degradation [2]: drying, chilling, freezing, pasteurization, irradiation, and high-pressure technology are the most common ones. The majority of them have many limitations: dehydration and change in the texture of the fruits (chilling/freezing), oxidation (chemical spoilage), loss of flavor and aroma (drying), loss of nutritional compounds (pasteurization and irradiation), huge amounts of plastics used (thermal sterilization and aseptic packaging), and high management costs (high-pressure techniques). In particular, freezing is the most used method. Unfortunately, it does not preserve fruits from microbial contamination and it is also the technique with more contraindications [3].

To extend the shelf life of berries (and fruits in general), different approaches have been explored in recent years, bringing innovations alongside the previous techniques. Indeed, technological advances allow the creation of plasmas (e.g., electric or electromagnetic fields on gases) [4,5] at atmospheric pressure, with or without ultraviolet (UV) rays. However, although the microbiological results are promising, these treatments damage cells, also at the DNA level [4], degrading the food. Moreover, advanced techniques of freezing have been implemented, such as the isochoric method [6]. This new method of freezing can preserve food without causing damage due to ice-crystal formation inside it, although it can generate hydrostatic pressures inside the processing chamber, deteriorating the food quality [7].

Moreover, the treatment of plants with chemical substances [8] has been considered: again, a decrease in bacteria has been demonstrated, but with concomitant degeneration in fruit properties. Other studies concentrated on creating biofilms [9,10] to cover berries’ surfaces. This approach is the most promising, because edible coatings, created with natural compounds, could act against the bacterial flora without modifying the organoleptic and antioxidant properties of the fruit. Unfortunately, at the moment there are not many “green” alternatives implemented in fruit industries [11,12]. This is due to the cost of nanotechnologies and to a lack of regulatory prescriptions about the use of nanocarriers in fruits [13].

Given this background, our work aimed to find a possible natural coating for berries which could preserve them from degradation without modifying their flavor and antioxidant properties. The present study proposes a treatment for freshly picked berries based on a natural compound, namely, curcumin. Curcumin is a natural polyphenolic compound from the spice turmeric (*Curcuma longa* L.) [14,15]. Being insoluble in water, curcumin should be solubilized by an inclusion complex to be delivered to treated samples.

It is worth noting that β-cyclodextrin is a good solubilizing agent, and previous research [16,17] has shown its capability to solubilize curcumin. Moreover, it is a safe component in medical and non-medical areas [18].

Previous experiences [19] showed that the β-cyclodextrin-curcumin complex is effective and safe for food applications and that curcumin preserves its valuable properties, e.g., it is a widely used antioxidant and exhibits a wide spectrum of antimicrobial activity per se [20]. Being a natural photosensitizer, when irradiated with visible light with a wavelength around 460–470 nm (blue light) or light in the ultraviolet range, curcumin causes massive inactivation of a broad range of food-related microorganisms [21]. Previous experiments on three bacterial strains, one Gram-negative (*Escherichia coli* ATCC 25922) and two Gram-positive (*Staphylococcus aureus* ATCC 29213 and *Enterococcus faecalis* ATCC 29212), showed a huge reduction in the curcumin minimum inhibitory concentration (MIC) following 3 h of LED irradiation in the Gram-positive species [19,22]. However, in these experiments, no statistical analyses were performed, and no rigorous tests were carried out regarding the flavor/taste of berries.

Photodynamic inactivation (PDI) is mainly due to physical processes elicited by the excitation of photosensitizers upon photo-absorption, which, interacting with molecular oxygen, generate reactive oxygen species (ROS) [21]. Following ROS production, nearby microorganisms undergo non-specific, highly cytotoxic oxidation reactions [21]. However, such oxidative processes could have a negative impact on the sensory or antioxidant properties of fruits. A similar effect on fruit taste might be exerted also by the solvent in which the curcumin (insoluble in water) has to be solubilized, i.e., β-cyclodextrin.

The aim of this work was, therefore, to assess the effectiveness of an antibacterial treatment with curcumin in β-cyclodextrin, photoactivated by blue light. In addition, the preservation of the odor and antioxidative properties of the berries were investigated. To achieve these goals, the experiments were performed in triplicate, and ad hoc tests and instruments were used. Various similar experiments on different fruits [23,24,25,26,27], vegetables [28], and fresh-cut fruits [29,30,31] had already been performed with success. This study differs from the previous ones in terms of both the composition and purpose of the coating used. Its focus was on the microbiological aspect rather than on the appeal and palatability of the fruit to the consumer (testing color, texture, consistency, and acidity). These important tests will be performed in further experiments to tune and optimize the treatments in order to attain the best microbiological response.

## 2. Materials and Methods

### 2.1. Materials and Chemicals

Unless otherwise stated, the materials employed were purchased from Sigma-Aldrich (St. Louis, MO, USA). The Β-cyclodextrin used was a gift from Roquette (Roquette Freres, Lestrem, France). Curcumin from Curcuma longa was purchased from Sigma Aldrich. Tripticase Soy Agar (TSA) plates for the microbiological essays were obtained from Liofilchem^®^.

The instruments used were: a spectrophotometer: Duo Beckman; Centrifuge: Rotofix 32A Hettich Centrifugen; a lux meter: mod. LMT B500 s/n 0180233 (photocell mod. P30SC0, s/n 287032), LMT Lichtmesstechnik GmbH; and an ultrasonic bath: Bandelin Sonorex Digitec; Bandelin Electronic GmbH & Co. KG, Berlin, Germany.

### 2.2. The Setting of the Experiments

The study was performed on strawberries and blueberries, separately treated in three experiments. All the experiments focused on the antimicrobial effects of the curcumin and its photoactivation, but in order to investigate separately the impact of the curcumin concentration and that of multiple irradiations, two more experiments were performed.

All the berries were bought from local markets in summer 2022. They were not frozen or subjected to any other type of preservation procedures.

In the first experiment (E1), strawberries were divided into 4 experimental groups:

1. Berries without treatment (CTR);
2. Berries sprayed with β-cyclodextrin solution (CI);
3. Berries sprayed with a solution of 0.5 mg/mL curcumin in β-cyclodextrin (CICU1);
4. Berries sprayed with a solution of 0.5 mg/mL curcumin in β-cyclodextrin and exposed to blue light for 3 h (CICUB1) after 1 h of incubation.

In the second experiment (E2), blueberries were divided into 4 groups:

1. Berries without treatment (CTR);
2. Berries sprayed with a solution of 1 mg/mL curcumin in β-cyclodextrin (CICU2);
3. Berries sprayed with a solution of 1 mg/mL curcumin in β-cyclodextrin and exposed to blue light for 3 h (CICUB2) after 1 h of incubation;
4. Berries prepared as CICUB2 and illuminated for 3 h every day for three days (CICUBm).

In the third experiment (E3), blueberries were divided into 5 groups:

1. Berries without treatment (CTR);
2. Berries sprayed with β-cyclodextrin solution (CI);
3. Berries sprayed with a solution of 1 mg/mL curcumin in β-cyclodextrin (CICU2);
4. Berries sprayed with a solution of 1 mg/mL curcumin in β-cyclodextrin and exposed to blue light for 3 h (CICUB2) after 1 h of incubation;
5. Berries prepared as CICUB2 and illuminated for 3 h every day for three days (CICUBm).

After the treatments, all groups were stored at refrigeration temperature (4 °C).

All of the experimental systems can be considered closed systems, to which no nutrients were added and from which no metabolic waste products were removed. When nutrients were depleted, growth would stop and metabolic waste products would accumulate in the medium. To avoid this death phase, in all three experiments, visual and microbiological controls were used at the end of treatment (T0) and after one (T24), two (T48), and three (T72) days. In addition, a longer time would cause the fruits to rot.

Volatile compound analyses were performed after four days in the first and third studies, while such an analysis was not conducted for E2 due to management problems. Moreover, the fruit samples sprayed with two different concentrations of curcumin (CICU1 with 0.5 mg/mL and CICU2 with 1 mg/mL) and the same samples treated with 3 h LED irradiation (CICUB1 and CICUB2) were investigated for oxidation and quantitative detection of curcumin content.

**Figure 1:**
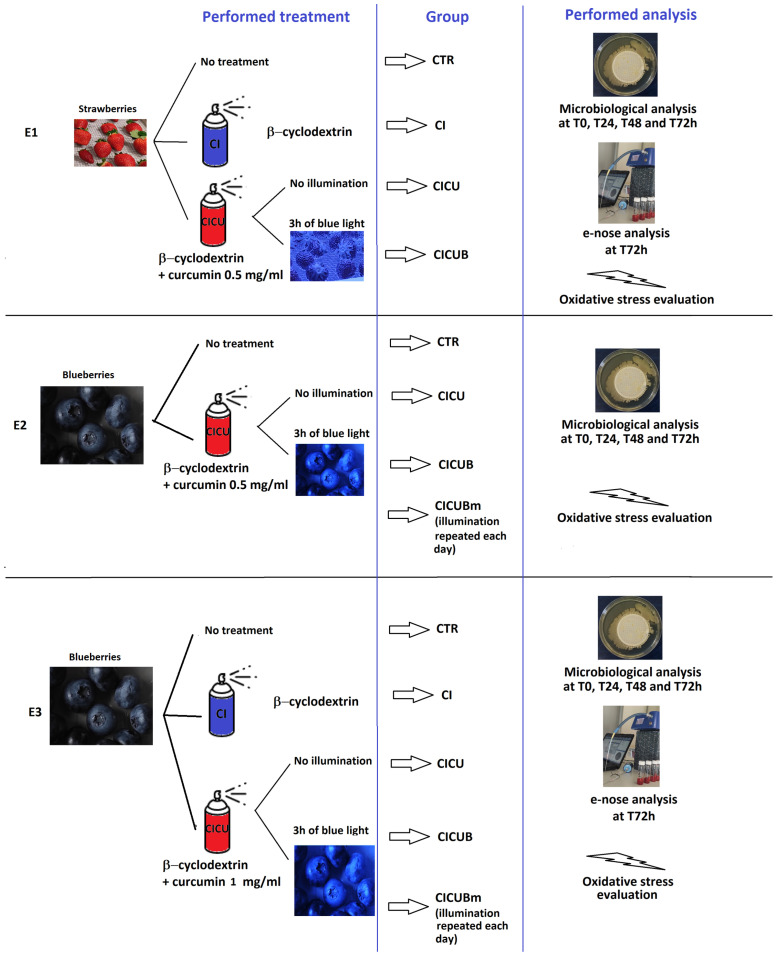
Sketch of the three experiments: in the first column, the performed treatments are summarized; in the second column, the names of the groups are associated with each procedure; and in the third column, the performed analyses are reported.

A summary of the three experiments is shown in Figure 1, while an explanation of the groups’ names is provided in Table 1.

### 2.3. Phase-Solubility Study of the β-Cyclodextrin/Curcumin Inclusion Complex

Phase-solubility studies were carried out using the method developed by Higuchi and Connors [32]. An excess amount of Curcumin was added to a series of aqueous solutions containing an increased concentration of β-cyclodextrin (0 to 70 mM) [33,34]. Mixtures were continuously shaken at 28 relative centrifugal force (RCF) for 72 h at room temperature in the dark. The resultant dispersions were then centrifuged for 25 min at 4025 RCF and the curcumin containing supernatant was separated and its curcumin concentration was determined using a spectrophotometer at a wavelength of 425 nm. The curcumin concentration was estimated on the basis of a previously obtained calibration plot. The apparent stability constants (*Ks*) of the curcumin–βCD complexes were calculated from phase-solubility diagrams according to the following equation:

(1) $$Ks=\frac{Slope}{S{}^{\circ}\;\left(1-slope\right)}$$

where the *slope* is obtained from the linear part of the phase-solubility diagram and *S*° is curcumin’s intrinsic solubility in the absence of cyclodextrin polymer.

### 2.4. Preparation of β-Cyclodextrin/Curcumin Inclusion Complexes

Based on the phase-solubility study, the inclusion complexes of curcumin and β-cyclodextrin were prepared at a molar ratio of 1:1. At first, 10 mg of curcumin was added to 10 mL of β-cyclodextrin aqueous solution at a concentration of 3 mg/mL (molar ratio: 1:1). The mixture was left under stirring for 48 h at room temperature in the dark. After 48 h, the resulting dispersion was centrifuged at 1370 RCF for 10 min. Finally, the supernatant, which contained the curcumin β-cyclodextrin complexes, was recovered for characterization and further experiments.

### 2.5. Preparation of the Curcumin-Complex Solution

The curcumin β-cyclodextrin complexes as freeze-dried powder were weighed and dissolved in water under magnetic stirring to prepare two aqueous solutions of 0.5 and 1 mg/mL of curcumin, respectively.

### 2.6. Curcumin Concentrations in Fruits after Treatment

To check whether the sprayed curcumin/βCD complexes effectively adhered to the fruit peel and were not degraded by LED light, the concentration of curcumin on berries was evaluated in three different cases: just after the spraying and after three hours of irradiation (with or without waiting for 1 h between spraying and light). This operation was performed for both berry types and for two curcumin concentrations (0.5 and 1 mg/mL). Curcumin concentration in the homogenized berries was determined using a spectrophotometer at a wavelength of 425 nm.

### 2.7. Photoactivation

**Figure 2:**
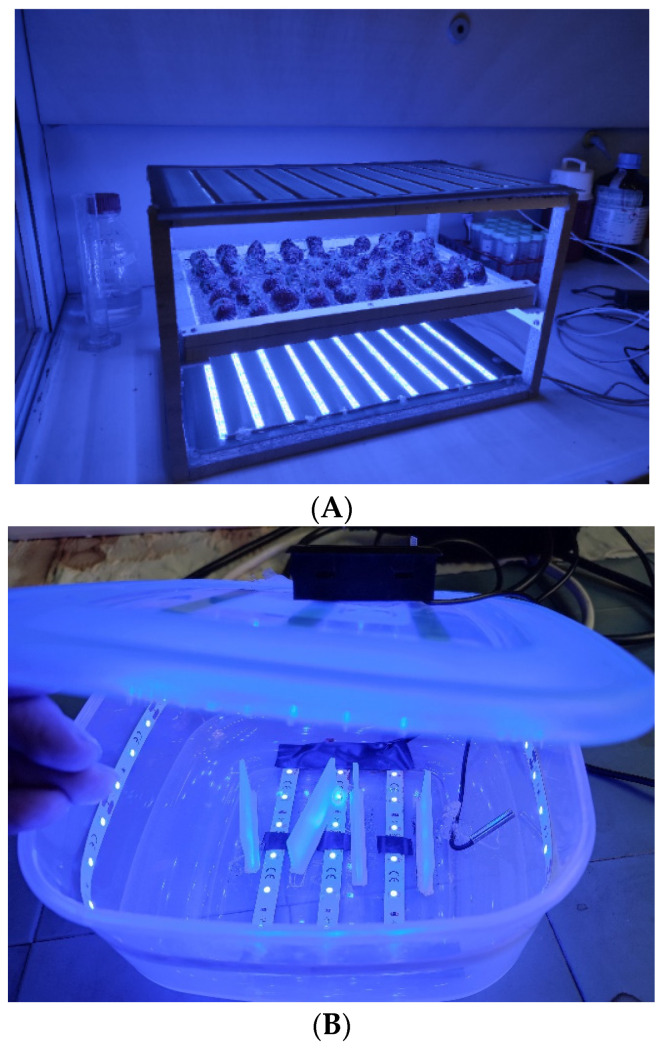
(**A**) On the left, the bigger structure was created to illuminate a maximum of 0.25 kg of berries with blue LED after curcumin spraying; berries were placed on the mid-frame grid, irradiated from both above and below. The structure was placed under a hood to avoid heating the berries during the three hours of illumination. (**B**) On the right, the smaller structure for in vitro experiments or a small number of berries can be seen. A Petri dish could be inserted on the supports to illuminate, from all the directions, berries or bacterial colonies.

Curcumin was photoactivated by blue LED (LES Flex Strips LEDYDEL IP64, Turin, Italy) with a wavelength of 465–470 nm. Berries were irradiated for 3 h from both sides. Two irradiation chambers were designed. The first one (Figure 2A) was used to irradiate up to approximately 0.25 Kg of berries lying on a grill at half height. Ten LED strips (27 leds each) were inserted on the base and ten on top, with a light flow per unit surface of 693 ± 83 lx. To prevent heating, the experiment was performed under a chemical hood with laminar flow.

The second irradiation chamber (Figure 2B) was smaller and used for the laboratory essays, i.e., oxidation tests, on small fruit samples (2–3 berries maximum). A Petri dish could be inserted inside on plastic supports; three LED strips (6 leds each) on the base, three on the top, and one on the side of the box generated a light flow per unit surface of 700 ± 25 lx which was perfectly comparable with the other.

The brightness measures were conducted with the lux meter mod. LMT B500 s/n 0180233 (photocell mod. P30SC0, s/n 287032), LMT Lichtmesstechnik GmbH.

### 2.8. Microbiological Analysis

After the photoactivation treatment, the counting of the colonies of microorganisms on the strawberries and blueberries was performed by the plate-counting method [35].

Initially, the strawberries were washed with 10 mL of sterile water, and 100 μL of washing water was spread on Trypticase Soy Agar (TSA) plates.

The strawberry and blueberry samples (5 g) were removed from the water, homogenized in 5 mL of sterile water, sequentially diluted, and inoculated on TSA. The bacterial counts for these samples are labelled as “Berry” at different times in the Section 3.

In addition, to quantify the bacteria on blueberries, after being exposed to the treatments, the fruits were placed in tubes containing 0.9% NaCl saline solution and sonicated (35 kHz; Bandelin Sonorex Digitec; Bandelin Electronic GmbH & Co., KG, Berlin, Germany) for 10 min to detach bacteria without affecting their viability [36]. After sonication, the fruits were removed, and the tubes were centrifuged at 1789 RCF for 10 min. The resulting pellets were resuspended and serially diluted in a saline solution. Each dilution was spread on TSA. The bacterial counts for these samples are labelled as “Peel” at different times in the Section 3. In other words, “Berry” refers to the counts of bacterial colonies present on the whole berries following homogenization, while “Peel” refers to those present on the peel only and which were detectable in water following washing.

All the agar plates were incubated at 37 °C for 24–48 h, allowing for optimal microbial growth, and then the total number of surviving colonies on each plate was counted.

### 2.9. E-Nose Analysis

The volatile compound analysis was performed with a commercial portable electronic nose (Win Muster Airsense Analytic Inc., Schwerim, Germany) equipped with 10 metal oxide semiconductors (MOSs) of different chemical compositions and thicknesses to provide selectivity towards volatile compound classes, as reported by Torri [37]. In particular, the sensors used were W1C (aromatic compounds), W5S (broad-range sensitivity, nitrogen oxides), W3C (Ammonia), W6S (Hydrogen, breath gases), W5C (Alkanes, aromatic compounds), W1S (Methane, environment), W1W (Sulphur compounds), W2S (Alcohols), W2W (Sulphur aromatic compounds), and W3S (high concentrations > 100 ppm, methane).

For each experiment and group, the berries were divided into ten samples of 10.0 ± 0.1 g and put in standard clear glass vials (40 mL) hermetically sealed with a PTFE/silicone septum and a screw cap. The vials were equilibrated at room temperature (26 °C) for 1 h and analyzed at the same temperature in standardized conditions. The device probe aspirated the volatile compounds from the vial headspace through the sensor array at 400 mL/min for 30 s. After sample measurement, the system was purged for 70 s at a flow rate of 600 mL/min with filtered air before the injection of the next sample to allow the instrument to re-establish a baseline.

Principal component analysis (PCA) of the data and hierarchical cluster analyses of the first three factors were performed using the XLSTAT statistical software package, version 2020.5 (Addinsoft, Paris, France).

### 2.10. Oxidative Stress Evaluation

#### 2.10.1. Malondialdehyde Test

Among oxidative stress assays, malondialdehyde (MDA) is the most widely used to evaluate the antioxidant activity of chemicals in lipid peroxidation systems. The oxidative breakdown of polyunsaturated fatty acids in acidic media produces MDA, which combines with Thiobarbituric Acid (TBA) to create the MDA—TBA adduct [38,39]).

The CTR, CTR + 3 h LED, CICU1, CICU2, CICUB1, and CICUB2 groups were tested. A control (treated only with Linolenic acid) was prepared by following the same procedure without a test sample. The testing samples, i.e., homogenized berries (100 µL), were added to 0.1 mL of distilled water, 0.2 mL of 4% sodium dodecyl sulfate (SDS), 1.5 mL of Phosphoric acid, and 100 µL of 0.1% linolenic acid. At the end, 1 mL thiobarbituric acid (TBA) solution was added to the mixture and heated at 100 °C for 45 min; later, it was cooled down in an ice bath, and 4 mL of 1-butanol was added. Then, the supernatant (TBA-MDA adduct) was extracted and analyzed using a UV-spectrophotometer (Duo Beckman) at 535 nm.

For the MDA standard curve, the same process was used, with the exception that instead of adding the 100 μL sample solution, the standard solution (1,1,3,3 tetra methoxy-propane) was added.

#### 2.10.2. DPPH Free Radical Scavenging Activity

The antioxidant activity of curcumin sprayed on blueberries and strawberries was investigated by determining its ability to scavenge 2,2-diphenyl-1-picrylhydrazyl (DPPH). The DPPH free radical scavenging activity of curcumin was evaluated also after 3 h of exposure to blue light.

DPPH is a lipophilic free radical that shows a purple color in solution and UV absorption at a wavelength of 517 nm. When DPPH undergoes a redox reaction with an antioxidant, it turns yellow, with a decrease in absorbance at 517 nm.

To perform the DPPH free radical scavenging assays, 100 µL of each sample (i.e., homogenized berries sprayed with curcumin) was added to a DPPH methanolic solution (900 µL, 0.1 mM) and incubated at room temperature for 30 min in the dark. Absorbance was measured at 517 nm against a blank using a spectrophotometer. All experiments were performed in triplicate. The percentage of antioxidant activity was calculated according to the following formula: DPPH scavenging (%) = [(A DPPH—A sample)/A DPPH] × 100.

### 2.11. Statistical Analysis

The results of the repeated microbiological experiments (in triplicate) were reported as averages with standard deviations. Differences between observation times were studied with ANOVA, considering time and treatments as factors, while comparisons between curcumin concentrations and between oxidative tests were evaluated by *t*-tests.

The *p*-values were reported, considering a type I error of 5%. Calculations were performed using SAS^®^ Statistic Software v 9.4.

## 3. Results

### 3.1. Phase-Stability Study

The solubility of curcumin increases as the β-cyclodextrin level increases. The curcumin/βCD inclusion complex displays the A_L_ type of solubility phase profile, according to [32], indicating a linear increase in solubility as a function of β-cyclodextrin concentration. The resulting value of Kc = 834.4 M^−1^ indicated that curcumin was satisfactorily solubilized. The plot of the phase-solubility curve is reported in the Supplementary Materials, Figure S3.

### 3.2. Curcumin Concentrations in Fruits after Treatment

To determine whether the sprayed solution effectively adhered to the fruit peel and was not degraded by LED light, the concentration of curcumin on berries was evaluated in three different cases: just after the spray and after three hours of irradiation (with or without waiting for 1 h between spray and light). This operation was performed for both berry types and for two curcumin concentrations (0.5 or 1 mg/mL).

The results are shown in Table 2 as µg of curcumin on g of berry. *p*-values refer to the comparisons of non-irradiated and irradiated fruits, with and without a rest of 1 h. Note that the final concentration strictly depends on the width of the fruit’s surface. The sprayed solution covered only the surface of the berries and not the entire volume.

A larger curcumin concentration was found when berries were irradiated after 1 h of incubation after spraying, probably because of the curcumin penetration across the peel. This difference was statistically significant in the case of the 0.5 mg/mL concentration on blueberries and for both concentrations on strawberries, while the difference of 1 mg/mL concentration on blueberries was not significant. This was probably due to the existence of a maximum carrying capacity, which was reached in the case of blueberries.

Doubling the curcumin concentration in the sprayed liquid enhanced, as expected, the quantity of curcumin found per g of fruit.

### 3.3. Gain Analysis

**Figure 3:**
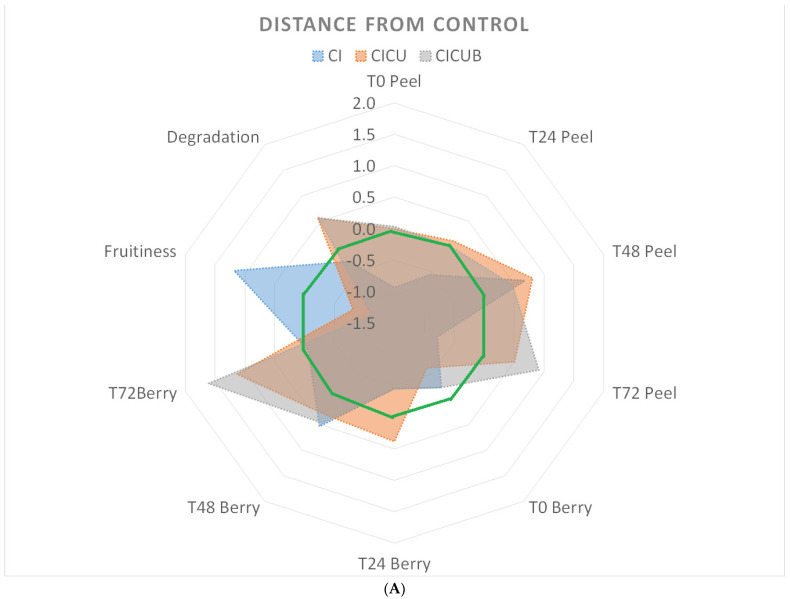

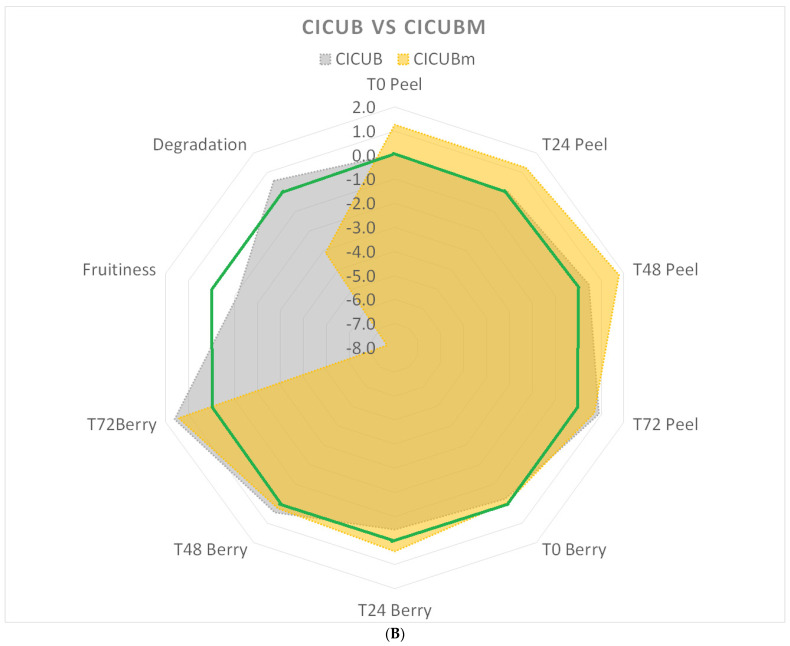
(**A**) (Top) Radar graph for CI (blue area), CICU (red area), and CICUB (grey area) in respect of CTR (green line). Both blueberries and strawberries were considered. All the points outside the green line indicate better behavior in respect of CTR, while inner points indicate worse behavior. (**B**) (Bottom) Radar graph for CICUB (grey area) and CICUBm (orange area) in respect of CTR (green line). All the points outside the green line indicate better behavior in respect of CTR, while inner points indicate worse behavior.

In Figure 3, all the results of the three experiments are summarized with radar graphs, while the numerical values and the relevant pictures are shown in the Supplementary Materials (Table S1). The radar charts were used to compare the three experiments, which differed from each other in terms of techniques and berries. Indeed, as explained in the next paragraphs, the radar graphs were made by normalizing the data with respect to the controls, making the data from different experiments comparable.

Microbiological analyses were divided into “Berry” and “Peel” ones. “Berry” refers to the analyses of smoothed berries, while “Peel” refers to the analyses of the water in which entire berries was washed. The tests highlighted a lower number of bacteria in the CICU, CICUB, and CICUBm groups, in particular in the E3 experiment, i.e., the one with a higher curcumin concentration (see Table S1 in the Supplementary Materials). The difference between groups was, in this case, statistically significant (*p*-values = 0.005 and 0.001, respectively, for washing water and smoothed berries). However, the differences between the E1 and E2 experiments, with the lowest curcumin concentration, were not statistically significant (*p* > 0.05). Probably, 0.5 mg/mL of curcumin is not sufficient to eliminate bacteria for a long time. The variations in time were all not significant.

Concerning the odor analysis, the first two main components of the PCA showed 75.38% and 84.87% of the variance of data, respectively, in the cases of strawberries and blueberries (Figures S1 and S2 in the Supplementary Materials show the biplots in output from the PCA analysis on top).

The two principal components were F1 (sensors: W1C, W3C, W6S, W1W, W2W, and W3S) and F2 (sensors: W5S, W5C, W1S, and W2S). A practical sense of these two components can be assigned, as in [40] in a similar case. F1 was widely impacted by methane, ammonia, and breath gases, so it indicated the “degradation” of berries. F2 recognized esters, i.e., the natural odor of berries, so it denoted “fruitiness”. These definitions are used in the radar graph (Figure 3A,B).

To summarize the results, the radar graphs in Figure 3A,B were produced by normalizing the data concerning the CTR group (green line). The area contained within the green line indicates the extent to which the treatment was less effective than no treatment, while the area that extends outside the green line quantifies the extent to which the treatment was more efficient than the control.

In particular, CTR berries (light blue circles at the top of Figures S1 and S2) were not distinguishable from CICUB (grey circles) ones in either of the experiments, showing an overlap of odors of the two groups. CI (blue circles) changed in the F1 component, probably because of the deodorant effect of β-cyclodextrin. The CICU group (red circles) was distinguishable from CTR in strawberries but not in blueberries. The CICUBm group had a strong F2 component, showing an alteration in odor. Considering the hierarchical clusters (the bottom of Figures S1 and S2), calculated on factor scores, an overlap of groups was seen in the case of strawberries. Indeed, two major clusters were found: the first one contained CTR, CICU, and fresh CICU (in prevalence), but also some CICUB samples; the second one contained CI and CICUB (in prevalence), but also CTR and CICU samples. In the case of blueberries, CICUBm created a very strong cluster, while the other groups were in another one. No significant changes in odor were therefore assessed in the two cases, except for the CICUBm group. Multiple irradiations probably significantly degrade the berries.

In conclusion, CI (blue area) did not exhibit any antimicrobial effect, but it is interesting to note with respect to the cluster created by the CI (blue) group that what enhanced the “fruitiness” of the samples was a natural deodorizing product that can mask the volatile compounds in berries. The addition of curcumin (CICU, red area), although it impacted the odor characteristics, improved the microbial resistance, especially at 72 h, both in the fruits and in the washing water.

LED blue light irradiation (CICUB, grey area) further enhanced the antimicrobial effects at all times.

The repetition of LED irradiation on different days (CICUBm, yellow area) did not radically modify the microbiological effectiveness but only worsened the fruit’s pleasantness.

### 3.4. Oxidative Stress Evaluation

**Figure 4:**
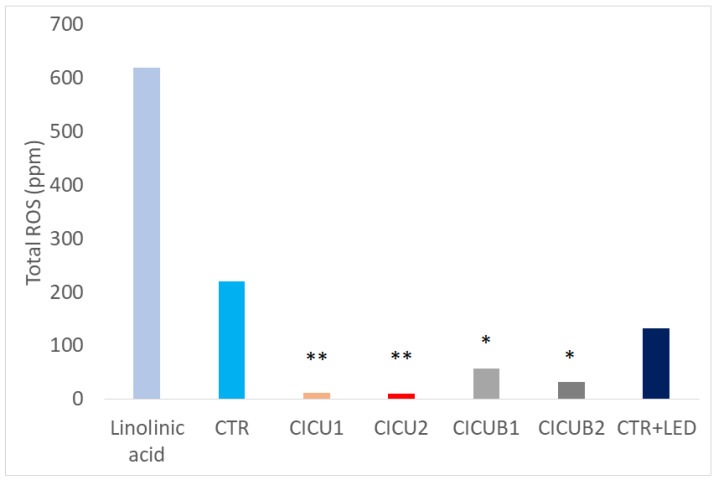
Oxidative stress in the five standard berry groups (CTR, CICU1, CICU2, CICUB1, and CICUB2; see Table 1) compared with the standard control (linolenic acid) and berries + blue LED. Both the treatments (grey bars, i.e., CICUB at 0.5 and 1 mg/mL) reduced the oxidative stress in respect of controls. Legend: * = significant difference, *p* < 0.05; ** = major significant difference, *p* < 0.0001; no marks = not significant.

In the MDA test, CICU1, CICU2, CICUB1, and CICUB2 showed less oxidative stress (12, 10, 57, and 32 PPM, respectively; *p* < 0.05) with respect to CTR (220 PPM) and irradiated CTR plus blue light (132 PPM) (see Figure 4). The DPPH test partially confirmed this result, showing a better antioxidant activity in the CICUB groups (*p* < 0.05 for Strawberries CICUB1, Strawberries CICUB2, and Blueberries CICUB2) and a worse one in the CICU groups (*p* < 0.0001 for Strawberries CICU1). Means and standard deviations of the measures are reported in Table 3. Indeed, the curcumin may have potentiated the antioxidative properties of the berries. Moreover, although LED irradiation was responsible for some oxidative stress, as expected, the curcumin (at both concentrations) counteracted such effects by keeping the oxidative stress lower than the control values.

## 4. Discussion

The results from this preliminary investigation confirm that curcumin is effective for berry preservation, reducing bacterial load without significantly affecting odor and antioxidant properties. Curcumin has already been used on fruits in various studies, using different carriers to deliver it. It was dissolved directly in juices [41] and incorporated in nanocarriers [42,43] and in films [44,45]. For example, a recent experiment by Chuacharoen [46] showed the effectiveness of curcumin-loaded zein nanoparticles in mango, while Zou [47] designed curcumin-loaded nanoparticles to produce a drink colorant with antibacterial properties. Curcumin, sprayed to produce covering bio-films, was successfully used in apple juice [48] (based on β-cyclodextrin/curcumin inclusion complex), avocados [49], and cocoons [50] (Polyvinyl alcohol), as well as in food simulants [51] (orange oil nanoemulsion). Chitosan-based nanocarriers for curcumin seem to delay maturity in litchis, strawberries, mangos, and plums [24]. Curcumin was also inserted in nanocrystal formulations [52] to preserve cooked clams, showing a reduction in bacteria and also an improvement in color. In general, curcumin has proven to be an excellent antibacterial in the food sector [44,53], but it is also used in the medical field [14,54,55] because of its antioxidant and anticancer properties.

Photodynamic activation further selectively enhances the antimicrobial effect. It is important to note that not only curcumin is used as a photoactivable material in food. Many natural substances are photosensitive [21] and already used in medicine, e.g., Porphyrin, Chlorins, and Phthalocyanines, in particular, chlorophyll [56], melatonin [8,57,58,59], hypericin [59,60], carbon [61], indocyanine [62], vitamins [63], and essential oils [64], have already been tested on food. Among them, curcumin seems to be one of the best because of its effect on both Gram-positive and Gram-negative bacteria, as already shown in dates, mango, litchis, and kumquats [23,24,25,26,27]; vegetables (tomatoes and lettuce) [28]; and fresh-cut fruits [29,30,31]; as well as in the experiments on blueberries and strawberries described here. PDI is a promising technique [65] in the food industry, having a low cost and easy application: in fact, a few LED strips are sufficient to photoactivate large quantities of fruit. However, it has not yet been fully applied on the market, due to the difficulty of translating laboratory results to the industrial chain and also to possible drawbacks. Indeed, photoactivation could lead to heat transfer [66] in food, which can cause deterioration. In the present paper, degradation was seen in the CICUBm groups, due to multiple irradiations. Another problem is managing light radiation for a long period (at least 1 h) and for large quantities of material. Indeed, highly perishable foods, such as meat and some types of fruit and vegetables, are processed in shorter times and stored in ways that are not suitable for PDI.

The use of β-cyclodextrin/curcumin inclusion complexes showed that they were able to adhere to the fruit peel and to act as an effective photosensitizer to induce PDI after 3 h of LED blue-light irradiation. The procedure did not worsen the odor properties of the treated fruits. Although the LED light, as expected, produced significant oxidative products when applied to untreated berries, the presence of the β-cyclodextrin/curcumin inclusion complexes reduced the impact of ROS, preserving the natural antioxidant properties of the berries mainly conferred by anthocyanins [67]. Moreover, the treatment seemed more effective in blueberries than strawberries. This was probably due to the texture of the berries: indeed, strawberries have a less smooth surface, which is more difficult to spray uniformly. Moreover, they are more delicate than blueberries.

A further improvement of the antimicrobial effects could be obtained by repeating the LED illumination daily three times, but the berries’ odor properties significantly worsened. Moreover, multi-illuminations could be difficult to implement in the supply chain. Indeed, the whole berry surfaces should be irradiated to blue light for the treatment to be effective, while berries are boxed or at least crowded during both transport and storage in markets.

Concerning the fruits’ presentation to consumers, the odor analysis showed no differences between the control and CICUB groups, while degradation was shown after multiple irradiations. No texture or color tests were performed, but a visual comparison highlighted no significant differences.

The assessment of curcumin concentration after treatment was also interesting, showing that it remained on the berries’ surfaces, performing its antibacterial effect for a long time. A larger concentration in the solution or a slower release, e.g., by curcumin incorporation in more long-lasting complexes, would keep on controlling the bacterial concentration on fruits, possibly prolonging berry shelf life.

Another important result came from the antioxidant tests. Considering that photoactivation should generate ROS, the antioxidant properties of berries could be degraded by this procedure. However, our tests showed that antioxidant properties were preserved and in some cases improved, as already reported in [24].

All the components of the solution are natural and safe, without contraindications [18,68]. Moreover, the complex is classified as a macroscopic solution and not as a nanoformulation, the latter being regulated by the European Food Safety Authority (EFSA) in certain cases because they could carry some risk to health [69].

Other investigations are in progress to enhance the effectiveness and feasibility of the procedure. In particular, lowering the illumination time by LED would make the treatment more usable by companies: the current production chain cannot allow for a three-hour standstill of the product to photoactivate the curcumin, and the treatment time should be reduced to under one hour to be applicable. We are exploring several possibilities, e.g., curcumin photoactivation concomitant spraying or the usage of properly designed nanostructures to slowly release curcumin at a concentration that is effective against microorganisms to bypass the need for long-time illumination.

## 5. Conclusions

This study investigated the role of curcumin with or without photoactivation on berries’ shelf life. Three experiments were conducted with different curcumin concentrations and different irradiation procedures. In particular, 0.5 and 1 mg/mL of curcumin in β-cyclodextrin and one or multiple irradiations with blue light were explored.

In this preliminary work, the major limitation is the short observation time (3 days), due to the perishability of the berries and the closed system used for the microbiological tests. However, the treatment was effective despite this, showing it to be a promising preservation method, especially for blueberries.

Many tests were performed on the stability and the adhesive properties of the solution, its antioxidant properties, and the preservation of organoleptic properties, assuring the good performance of the treatment. The short- and long-time toxicity of the treatment with respect to consumers’ health was not explored, but the safety of all the used compounds is well-proven and they are authorized by the EFSA.

Given these promising results, new experiments are planned for new coating carriers, such as nanocrystals and nanobubbles, which should be able to release curcumin for a longer time. Particular attention will be paid to other important variables not tested in this preliminary work, such as texture, color, acidity, and the ability of the coating to delay the maturation of berries.

In conclusion, the tested dilution of curcumin in β-cyclodextrin, followed by photoactivation, has the requisites to be explored in future experiments as a natural, safe, and efficient coating for berry preservation.

## Figures and Tables

**Table 1:** Summary of the groups used in the three experiments.

Group Name	Explanation	Related Experiment
CTR	Berries without treatment	E1, E2, and E3
CI	Berries sprayed with a solution of β-cyclodextrin	E1, E2, and E3
CICU1/CICU2	Berries sprayed with a solution of β-cyclodextrin and 0.5/1 mg/mL of curcumin	E1 (CICU1), E2, and E3 (CICU2)
CICUB1/CICUB2	Berries sprayed with a solution of β-cyclodextrin and 0.5/1 mg/mL of curcumin and illuminated by blue LED for 3 h	E1 (CICUB1), E2, and E3 (CICUB2)
CICUBm	Berries sprayed with a solution of β-cyclodextrin and 1 mg/mL of curcumin, illuminated by blue LED for 3 h each day (for three days)	E2 and E3

**Table 2:** Results of the experiments on curcumin concentrations on berries after treatment. *p*-values refer to the comparisons of non-irradiated (first row) and irradiated fruits, with (third row) and without (second row) a rest of 1 h. The analysis was performed considering both blueberries and strawberries at 0.5 and 1 mg/mL of initial concentration.

	Blueberries				Strawberries			
	Spray 0.5 mg/mL	*p*-Value	Spray 1 mg/mL	*p*-Value	Spray 0.5 mg/mL	*p*-Value	Spray 1 mg/mL	*p*-Value
Curcumin + β-cyclodextrin spray	0.69 ± 0.01 µg/g		0.92 ± 0.30 µg/g		0.35 ± 0.01 µg/g		0.39 ± 0.01 µg/g	
Curcumin + β-cyclodextrin spray, blue light with LED 3 h	0.63 ± 0.16 µg/g	0.29	1.16 ± 0.05 µg/g	0.14	0.33 ± 0.01 µg/g	0.03	0.47 ± 0.03 µg/g	0.004
Curcumin + β-cyclodextrin spray, 1 h rest, blue light with LED 3 h	1.00 ± 0.05 µg/g	<0.0001	1.09 ± 0.08 µg/g	0.22	0.49 ± 0.02 µg/g	<0.0001	0.82 ± 0.02 µg/g	<0.0001

**Table 3:** DPPH scavenging (%) of treated/not treated blueberries and strawberries. Statistical comparisons were made between CTR and the other groups, per fruit. Legend: * = significant difference, *p* < 0.05; ** = major significant difference, *p* < 0.0001; no marks = not significant.

DPPH Scavenging %	Strawberries	Blueberries	Only Curcumin
CTR	78.3% ± 3.01	75.9% ± 3.05	50.0% ± 2.01
CTR + blue LED	78.4% ± 2.55	78.6% ± 1.22	88.9% ± 2.14 **
CICU1 (0.5 mg/mL)	71.1% ± 1.09 **	77.3% ± 1.88	
CICUB1 (0.5 mg/mL)	82.8% ± 3.11 *	76.4% ± 3.44	
CICU2 (1 mg/mL)	76.1% ± 2.08	72.7% ± 2.33	
CICUB2 (1 mg/mL)	84.4% ± 1.09 *	82.8% ± 3.66 *	

## Data Availability

Data is contained in Supplementary Materials, see https://doi.org/10.5281/zenodo.7939981 accessed on 7 December 2022.

## Supplementary Materials

The following supporting information can be downloaded at: https://www.mdpi.com/article/10.3390/foods12102038/s1.

## Abbreviations

βCD	β-cyclodextrin
EFSA	European Food Safety Authority
MIC	Minimum inhibitory concentration
MOS	Metal oxide semiconductors
Mt	Million tons
LED	Light-emitting diode
PCA	Principal component analysis
PDI	Photodynamic inactivation
RCF	Relative centrifugal force
ROS	Reactive oxygen species
TSA	Trypticase Soy Agar
UV	Ultraviolet
