# Supplementary material for: Combining Blue Light and Yellow Curcumin to Obtain a “Green” Tool for Berry Preservation against Bacterial Contamination: A Preliminary Investigation
Source: Foods. 2023 May 18;12(10):2038. doi: 10.3390/foods12102038 (PMC10217088; doi:10.3390/foods12102038)
Supplement: Supplementary file 1 [file foods-12-02038-s001.zip › foods-2326672-supplementary.docx]

Supplementary Materials

**Table S1.** Results of the three experiments. Values are in logarithms of the number of bacterial colonies at T0 and after 24, 48, and 72 h, for both peel and smoothed berries.

| **Experiment** | **Group** | **Washing water (peel)** | | | | **p-value** | **Smoothed berry (5 g)** | | | | **p-value** |
| --- | --- | --- | --- | --- | --- | --- | --- | --- | --- | --- | --- |
|  |  | **0** | **24** | **48** | **72** |  | **0** | **24** | **48** | **72** |  |
| **Strawberries (1 mg/ml)** | CTR | 4.4 | 3.3 | 3.5 | 4.6 | 0.66 | 2.3 | 2.8 | 2.9 | 2.6 | 0.66 |
|  | CI | 4.6 | 2.7 | 3.9 | 5.1 |  | 1.9 | 2.8 | 2.1 | 3.0 |  |
|  | CICU | 4.2 | 4.0 | 4.2 | 4.0 |  | 3.0 | 2.0 | 3.2 | 2.2 |  |
|  | CICUB | 4.1 | 3.6 | 4.1 | 3.4 |  | 2.2 | 2.5 | 2.7 | 2.0 |  |
| **Blueberries (1 mg/ml)** | CTR | 5.4 | 5.3 | 6.9 | 6.0 | 0.94 | 1.5 | 3.1 | 3.3 | 5.1 | 0.60 |
|  | CICU1 | 4.7 | 3.5 | 3.3 | 4.6 |  | 2.9 | 2.1 | 1.6 | 1.6 |  |
|  | CICUB1 | 5.8 | 4.7 | 7.0 | 4.8 |  | 2.1 | 4.1 | 2.8 | 1.6 |  |
|  | CICUBm1 | 2.9 | 3.2 | 4.4 | 4.8 |  | 1.4 | 1.6 | 2.8 | 2.9 |  |
| **Blueberries (2 mg/ml)** | CTR | 3.3 | 3.7 | 5.8 | 3.9 | 0.003 | 2.1 | 2.0 | 3.1 | 3.1 | 0.01 |
|  | CI | 5.0 | 5.4 | 4.1 | 4.9 |  | 3.0 | 3.0 | 2.9 | 2.9 |  |
|  | CICU2 | 4.2 | 4.3 | 5.6 | 3.8 |  | 2.5 | 2.4 | 3.2 | 2.6 |  |
|  | CICUB2 | 3.2 | 3.9 | 3.7 | 3.4 |  | 2.3 | 2.7 | 2.4 | 2.5 |  |
|  | CICUBm2 | 3.3 | 3.4 | 4.7 | 3.6 |  | 2.4 | 2.7 | 3.0 | 2.5 |  |

Table S2 Sensors' description of e-nose (from [37])

| Name of the sensor | Sensitive substances | Reference [37] |
| --- | --- | --- |
| W1C | Aromatic compounds | Toluene, 10 ppm |
| W5S | Broad range sensitivity, nitrogen oxides | NO_2_, 1 ppm |
| W3C | Ammonia | Propane, 1 ppm |
| W6S | Hydrogen, breath gases | H_2_, 100 ppb |
| W5C | Alkanes, aromatic compounds, less polar compounds | Propane, 1 ppm |
| W1S | Methane (environment) | CH_3_, 100 ppm |
| W1W | Sulfur compounds, terpenes, limonene, pyrazine | H_2_S, 1 ppm |
| W2S | Alcohols, a broad range of aromatic compounds | CO, 100 ppm |
| W2W | Sulfur aromatic compounds | H_2_S, 1 ppm |
| W3S | Reacts on high concentrations >100 ppm, methane | CH_3_, 10CH_3_, 100 ppm |

**Figure S1**. *Top*: PCA analysis of strawberries from e-nose data. Each dot represents a different sample. Different colors represent different treatment groups. *Bottom*: hierarchical cluster based on factor scores.

**Figure S2**. *Top*: PCA analysis of blueberries from e-nose data. Each dot represents a different sample. Different colors represent different treatment groups. *Bottom*: hierarchical cluster based on factor scores.


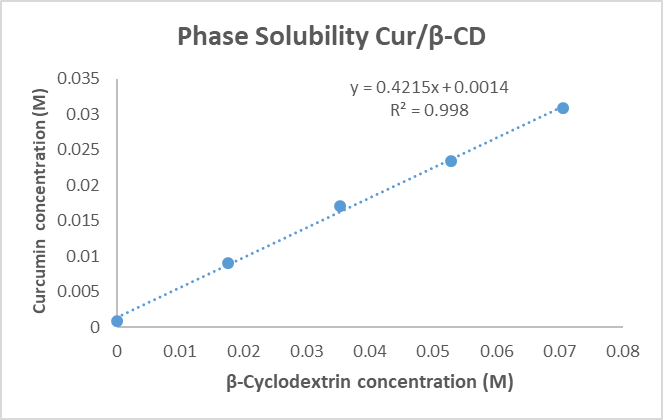


Figure S3 Phase solubility diagram of curcumin
